# HOME BUT HOMEBOUND AFTER TBI: PREVALENCE AND CORRELATES OF HOMEBOUNDNESS AFTER TBI

**DOI:** 10.1093/geroni/igad104.2786

**Published:** 2023-12-21

**Authors:** Raj Kumar, Katherine Ornstein, Shannon Juengst, Jennifer Reckrey, Amy Wagner, Laura Dreer, Kirk Lercher, Kristen Dams-O’Connor

**Affiliations:** Icahn School of Medicine at Mount Sinai, New York City, New York, United States; Johns Hopkins University, Baltimore, Maryland, United States; TIRR Memorial Hermann, Houston, Texas, United States; Icahn School of Medicine at Mount Sinai, New York City, New York, United States; University of Pittsburgh, Pittsburgh, Pennsylvania, United States; University of Alabama at Birmingham, Birmingham, Alabama, United States; Kessler Institute for Rehabilitation, West Orange, New Jersey, United States; Icahn School Of Medicine At Mount Sinai, New York City, New York, United States

## Abstract

Discharge home following post-acute care for traumatic brain injury (TBI) has historically been viewed as an unequivocally positive post-treatment outcome. Payors incentivize hospitals to discharge patients home and minimize readmissions. A historically overlooked topic in post-acute TBI care is homeboundness, which is the state where an individual rarely or never leaves home. Homeboundness is associated with premature mortality and poor physical and mental health. Our study investigates the prevalence and correlates of homeboundness among 6,595 non-institutionalized adults (mean age=42, range 16-99) who received inpatient rehabilitation for TBI in the TBI Model Systems National Database, a multicenter prospective cohort study. Our definition of homebound was based on self-reported number of days participants get out of the house in a typical week. We found that 14.2% of participants were completely homebound (never left the home) or partially homebound (left the home 1-2 times/week) 1-year after TBI. Using multivariable regression, we found the following variables were significantly associated with being completely/partially homebound at 1-year post-injury: older age, < college degree, Medicaid insurance, living alone and rural settings, not driving independently, and having functional needs for walking, upper body dressing, bowel and bladder, problem solving, and social interactions. These variables together had an area-under-the-curve of 0.81 distinguishing homeboundness. Our study elucidates an invisible subgroup of non-institutionalized adults living with TBI in the community who require ongoing support services or other follow-up care to improve functional status and participation. Implications for community integration will be discussed.

